# Induced Terpene Accumulation in Norway Spruce Inhibits Bark Beetle Colonization in a Dose-Dependent Manner

**DOI:** 10.1371/journal.pone.0026649

**Published:** 2011-10-19

**Authors:** Tao Zhao, Paal Krokene, Jiang Hu, Erik Christiansen, Niklas Björklund, Bo Långström, Halvor Solheim, Anna-Karin Borg-Karlson

**Affiliations:** 1 Ecological Chemistry Group, Department of Chemistry, Royal Institute of Technology, Stockholm, Sweden; 2 Norwegian Forest and Landscape Institute, Ås, Norway; 3 Department of Ecology, Swedish University of Agricultural Sciences, Uppsala, Sweden; Seoul National University, Republic of Korea

## Abstract

**Background:**

Tree-killing bark beetles (Coleoptera, Scolytinae) are among the most economically and ecologically important forest pests in the northern hemisphere. Induction of terpenoid-based oleoresin has long been considered important in conifer defense against bark beetles, but it has been difficult to demonstrate a direct correlation between terpene levels and resistance to bark beetle colonization.

**Methods:**

To test for inhibitory effects of induced terpenes on colonization by the spruce bark beetle *Ips typographus* (L.) we inoculated 20 mature Norway spruce *Picea abies* (L.) Karsten trees with a virulent fungus associated with the beetle, *Ceratocystis polonica* (Siem.) C. Moreau, and investigated induced terpene levels and beetle colonization in the bark.

**Results:**

Fungal inoculation induced very strong and highly variable terpene accumulation 35 days after inoculation. Trees with high induced terpene levels (n = 7) had only 4.9% as many beetle attacks (5.1 vs. 103.5 attacks m^−2^) and 2.6% as much gallery length (0.029 m m^−2^ vs. 1.11 m m^−2^) as trees with low terpene levels (n = 6). There was a highly significant rank correlation between terpene levels at day 35 and beetle colonization in individual trees. The relationship between induced terpene levels and beetle colonization was not linear but thresholded: above a low threshold concentration of ∼100 mg terpene g^−1^ dry phloem trees suffered only moderate beetle colonization, and above a high threshold of ∼200 mg terpene g^−1^ dry phloem trees were virtually unattacked.

**Conclusion/Significance:**

This is the first study demonstrating a dose-dependent relationship between induced terpenes and tree resistance to bark beetle colonization under field conditions, indicating that terpene induction may be instrumental in tree resistance. This knowledge could be useful for developing management strategies that decrease the impact of tree-killing bark beetles.

## Introduction

Bark beetles (Coleoptera, Scolytinae) and their associated microorganisms are among the most economically important threats to conifer forests worldwide [1]–[3]. In Eurasia, the spruce bark beetle *Ips typographus* (L.) is the most serious killer of mature Norway spruce, *Picea abies* (L.) Karsten [4]. This beetle is associated with several phytopathogenic bluestain fungi, including *Ceratocystis polonica* (Siem.) C. Moreau which seems to play an important role in tree death following beetle attack [5]–[8]. Experimental inoculation with fungus has been routinely used as a substitute for beetle attack in studies of tree resistance to *I. typographus* [9]–[11].

Conifer resistance against bark beetles and associated fungi involves multiple anatomical and chemical defense mechanisms [12], [13]. Induced defense mechanisms that develop around each attack point include traumatic resin duct formation, accumulation of terpenes, changed phenolic composition and proliferation of polyphenolic parenchyma cells [13]–[18]. Induced defenses have been studied extensively and seem to play essential roles in conifer resistance against the bark beetle-fungus complex [13], [19], [20].

The ecological functions of induced defenses have been studied by manipulating tree defenses using different stressors, such as mechanical wounding, fungal inoculation, beetle attacks and more recently methyl jasmonate (MeJA) application. These stressors elicit diverse anatomical and chemical defense reactions in the bark and sapwood [21]–[26], and elicited trees have been demonstrated to be much more resistant to massive fungal inoculation [9], [10], [27], [28] and bark beetle colonization [29]. Some studies have attempted to correlate host defense reactions, such as induced resinosis, with resistance to attack [10], [16], [29]–[31], but the precise role of host defenses during beetle colonization is still unknown.

Conifer resin, a mixture of structurally diverse mono-, sesqui- and diterpenes, has been extensively studied in many conifer-bark beetle systems due to its conspicuous nature and multiple ecological functions [12], [32], [33]. Monoterpenes are important in bark beetle host colonization, since they are used as primary chemical cues and/or precursors for the beetle's aggregation pheromones [34], [35]. In addition, terpenes seem to be essential components of conifer resistance due to their physically repellent and chemically toxic properties [13], [32]. However, the evidence for a defensive role of terpenes is still largely indirect and rests on the fact that synthetic monoterpenes may be lethal to bark beetles [20], [36] and inhibit the growth of beetle-associated fungi in lab bioassays [37], and that induction of terpenes by e.g. MeJA or fungal inoculation reduces host colonization by bark beetles [29] and their associated fungi [10], [38]. More precise knowledge about the function of terpene accumulation under field conditions, such as which terpene concentrations are sufficient to inhibit bark beetle attack, is needed to more fully understand the role of terpenes in conifer resistance.

Our aim in the present study was to induce terpene production in Norway spruce trees and to quantify the relationship between terpene levels and colonization by *I. typographus* in individual trees. The experiment was conducted in an area with outbreak populations of *I. typographus* following a severe storm in 2005, offering an ideal opportunity to test tree resistance to bark beetle colonization under natural conditions.

## Results

### Terpene levels varied extensively between trees and increased with time since inoculation

**Figure 1:**
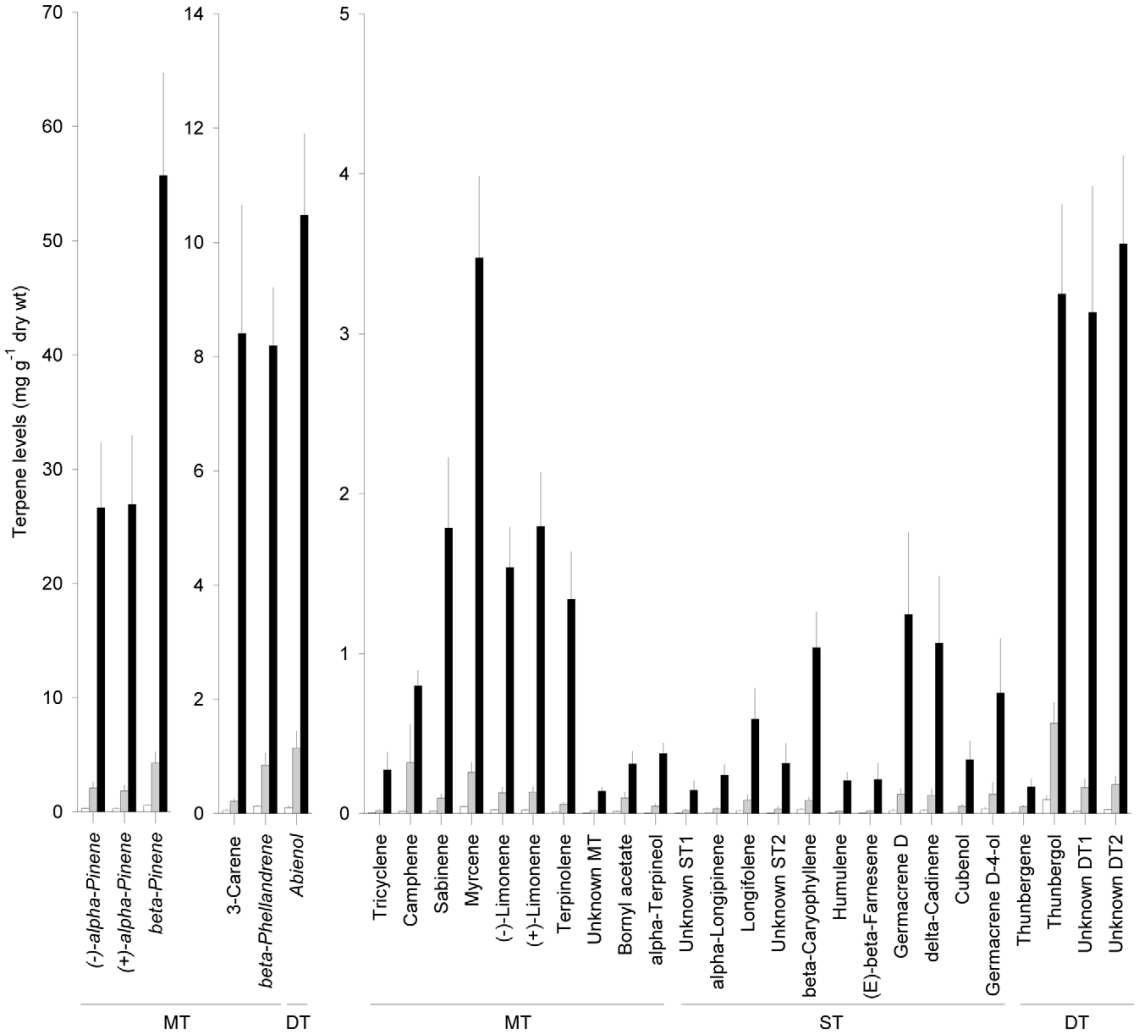
Levels of individual mono- (MT), sesqui- (ST) and diterpenes (DT) in untreated Norway spruce bark (white bars) and in bark close to inoculation sites with *Ceratocystis polonica* 14 days (grey bars) and 35 days (black bars) after inoculation. Data are expressed as means + 1 SE. n = 20 trees. All terpenes were significantly more abundant in inoculated bark at day 35 than in untreated bark (F_2,57_>7.21, *p*<0.0016) and a few compounds were significantly more abundant also at day 14 (labels in italics; F_2,57_>97.83, *p*<0.0001) (LSD test at *p* = 0.05 following ANOVA).

Phloem that had been induced with low densities of *C. polonica* inoculation had significantly increased levels of all analyzed terpenes relative to uninoculated control phloem. The increase was much stronger and more consistent at the latest sampling time (Fig. 1). Fourteen days after fungal inoculation levels of total mono-, sesqui- and diterpenes were 7, 6 and 9 fold higher, respectively, in the inoculated stem section than in the untreated section, and at day 35 the corresponding differences were 94, 52 and 101 fold.

**Figure 2:**
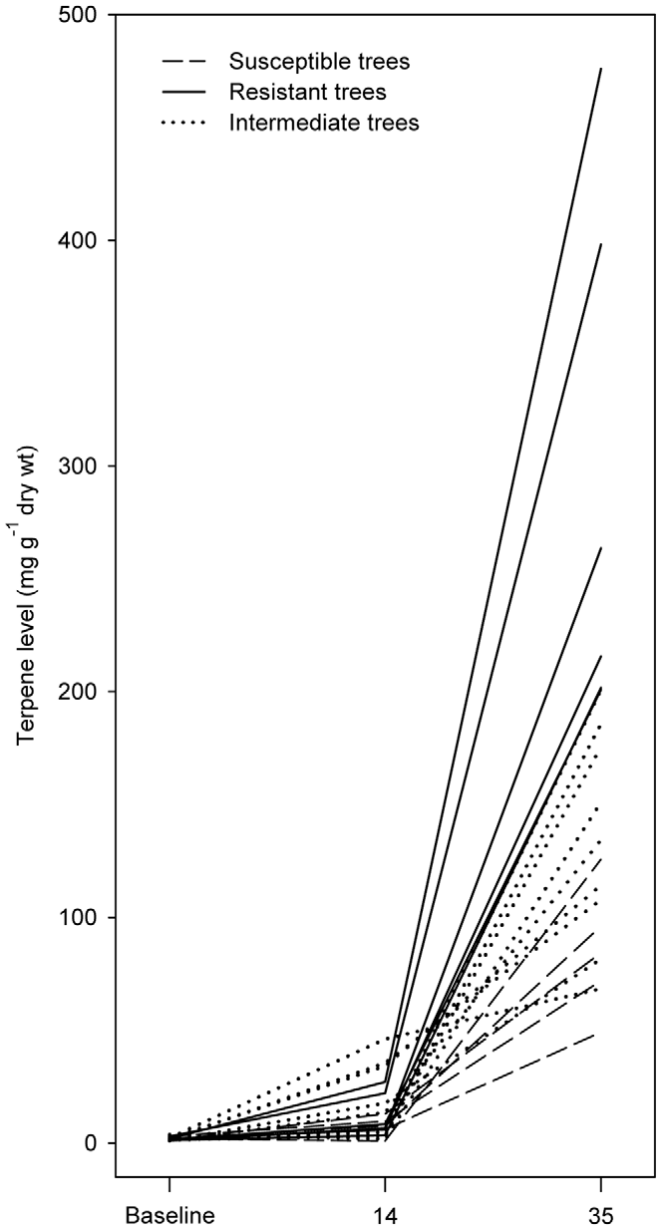
Terpene levels in the wound reaction zone in Norway spruce bark 14 and 35 days after *Ceratocystis polonica* inoculation and baseline levels in uninoculated bark on day 14 and 35. Based on a weighted average of the number of attacks and extent of gallery construction by *Ips typographus* trees were classified as susceptible (114.4±70.3 (SD) attacks and 1.5±1.0 m beetle galleries m^−2^ bark; n = 5) or resistant (1.1±2.6 beetle attacks and 0.02±0.04 m beetle galleries m^−2^ bark; n = 6). Intermediate trees had 31.6±16.8 attacks and 0.2±0.26 m beetle galleries m^−2^ (n = 9).

Induced terpene levels differed extensively between trees at both sampling times. Absolute terpene levels in the reaction zone of individual trees were 0.9–46.1 and 49.1–476.0 mg g^−1^ dry phloem at day 14 and day 35, respectively (Fig. 2). Compared to untreated phloem total terpene levels in individual trees had increased 1–29 fold on day 14 and 31–240 fold on day 35 after fungal inoculation.

The terpene response to fungal inoculation was more quantitative than qualitative, but for seven minor compounds (together constituting 9.9% of the total terpene amount in untreated phloem) the relative proportion was significantly higher in the reaction zone than in the untreated phloem (day 14: α-terpineol and abienol; day 35: (+)−3-carene, terpinolene, α-terpineol, an unidentified sesquiterpene and two unidentified diterpenes; F>3.21, *p*<0.05). The relative proportion of (+)−3-carene increased 5 to 52 fold in the reaction zone of 10 of the 20 experimental trees at day 35. Five minor compounds (together constituting 12.5% of total terpenes in untreated phloem) decreased in relative proportion in the reaction zone relative to untreated phloem (day 14: myrcene; day 35: β-phellandrene, (*E*)- β-caryophyllene, (-)-germacrene D-4-ol, and thunbergene; F>3.52, *p*<0.036).

The speed of terpene induction also varied between trees and with time since inoculation. Total terpene levels increased much slower the first two weeks after inoculation than during the succeeding three weeks (Fig. 2; t = −7.09, df = 19, *p*<0.0001; paired t-test of rate of increase in individual trees). Trees with high terpene levels at day 14 did not necessarily have high levels at day 35, and there was no significant correlation between terpene levels 14 and 35 days after inoculation (R^2^ = 0.0003, *p* = 0.94). The three trees that had the highest terpene levels in the reaction zone 14 days after inoculation had for example below average levels at day 35 (Fig. 2).

### Trees with high terpene levels had fewer attacks than trees with low terpene levels

**Figure 3:**
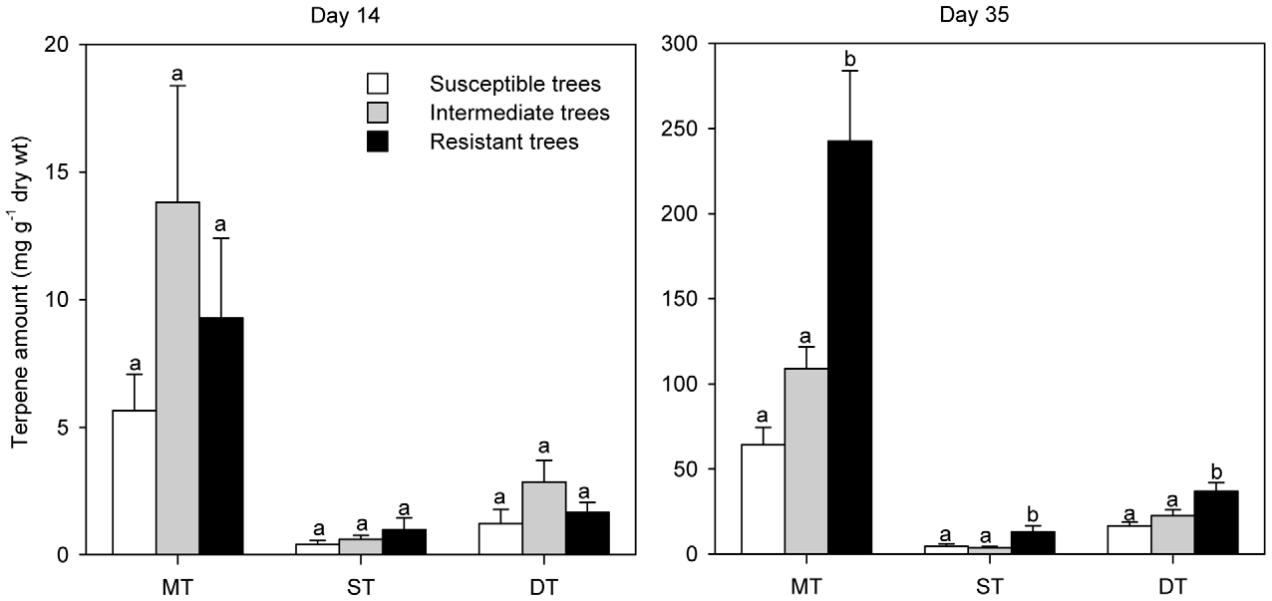
Total amounts of mono- (MT), sesqui- (ST) and diterpenes (DT) in the stem bark of susceptible (white), intermediate (grey) and resistant (black) Norway spruce trees (see **Figure 2** for details), 14 and 35 days after inoculation with the bluestain fungus *Ceratocystis polonica*. Data are expressed as means + 1 SE. Bars with different letters were significantly different by LSD test at *p* = 0.05 following ANOVA.

The six trees with least beetle colonization (0–6 beetle entrance holes and 0–0.09 m gallery m^−2^ bark surface) were considered resistant to attack, and the five trees with the most extensive beetle colonization (23–186 entrance holes and 0.36–3.0 m gallery m^−2^) were considered susceptible. The remaining nine trees were considered intermediate (8–57 entrance holes, 0–0.76 m gallery m^−2^). The beetle colonization level of individual trees was determined based on a normalized average of attack density and gallery length, where the value for each tree was expressed relative to the average for all trees. Resistant trees had significantly higher terpene levels in the reaction zone than intermediate and susceptible trees 35 days after fungal inoculation (Fig. 3; F_2,17_ = 14.89, *p* = 0.0002 for monoterpenes, F_2,17_ = 9.09, *p* = 0.002 for sesquiterpenes, F_2,17_ = 7.11, *p* = 0.006 for diterpenes). Mean concentrations of mono-, sesqui- and diterpenes in the reaction zones of resistant trees were 2.2 fold, 3.4 fold and 1.6 fold higher than in intermediate trees, and 3.8 fold, 2.9 fold and 2.2 fold higher than in susceptible trees. No significant differences between resistant and susceptible trees were found 14 days after inoculation for any terpene class (Fig. 3; F_2,17_<0.82, *p*>0.46).

### Terpene levels in the bark reduced beetle colonization in a dose-dependent way

**Figure 4:**
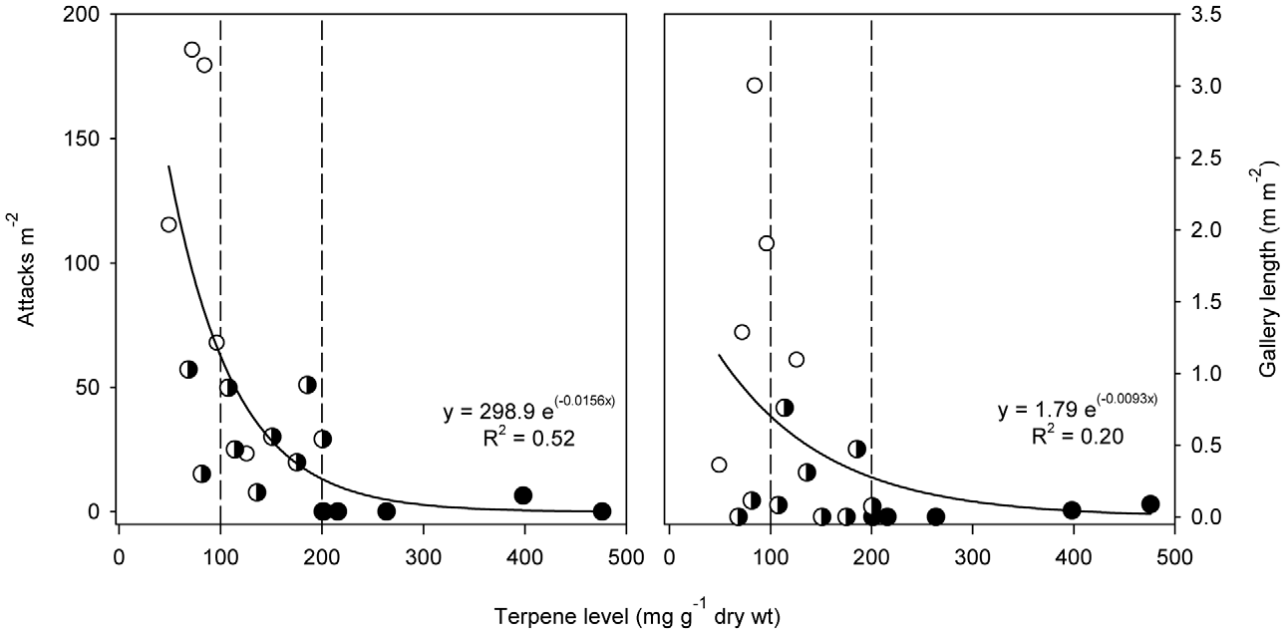
Attack densities (Left) and gallery lengths (Right) in 20 Norway spruce trees with different induced terpene levels in the bark. Chemical samples were taken from the reaction zone 35 days after inoculation with the bluestain fungus *Ceratocystis polonica.* Dotted lines show two potential thresholds in terpene levels (200 and 100 mg g^−1^ dry wt) with different inhibitive effects on beetle colonization. Solid lines show the fitting of a two-parameter single exponential decay function to the data. Trees were classified as resistant (black dots), susceptible (white dots) or intermediate (black and white dots) with respect to beetle colonization success (see Figure 2 for details).

There was a highly significant rank correlation between total terpene levels 35 days after inoculation and beetle colonization for individual trees (Spearman's rank correlation: r_s_ = 0.78, *p* = 0.0008), but there was no such correlation 14 days after inoculation (r_s_ = −0.08, *p* = 0.73). The relationship between terpene levels and beetle colonization was not linear, but followed an exponential decay function and seemed to involve two thresholds (Fig. 4). Above a high threshold concentration (∼200 mg terpene g^−1^ dry phloem) trees largely escaped beetle attacks, as there were few entrance holes and short galleries in such trees. Above a low threshold concentration (∼100 mg terpene g^−1^ dry phloem) the trees had moderate attack density and gallery construction (Fig. 4).

**Figure 5:**
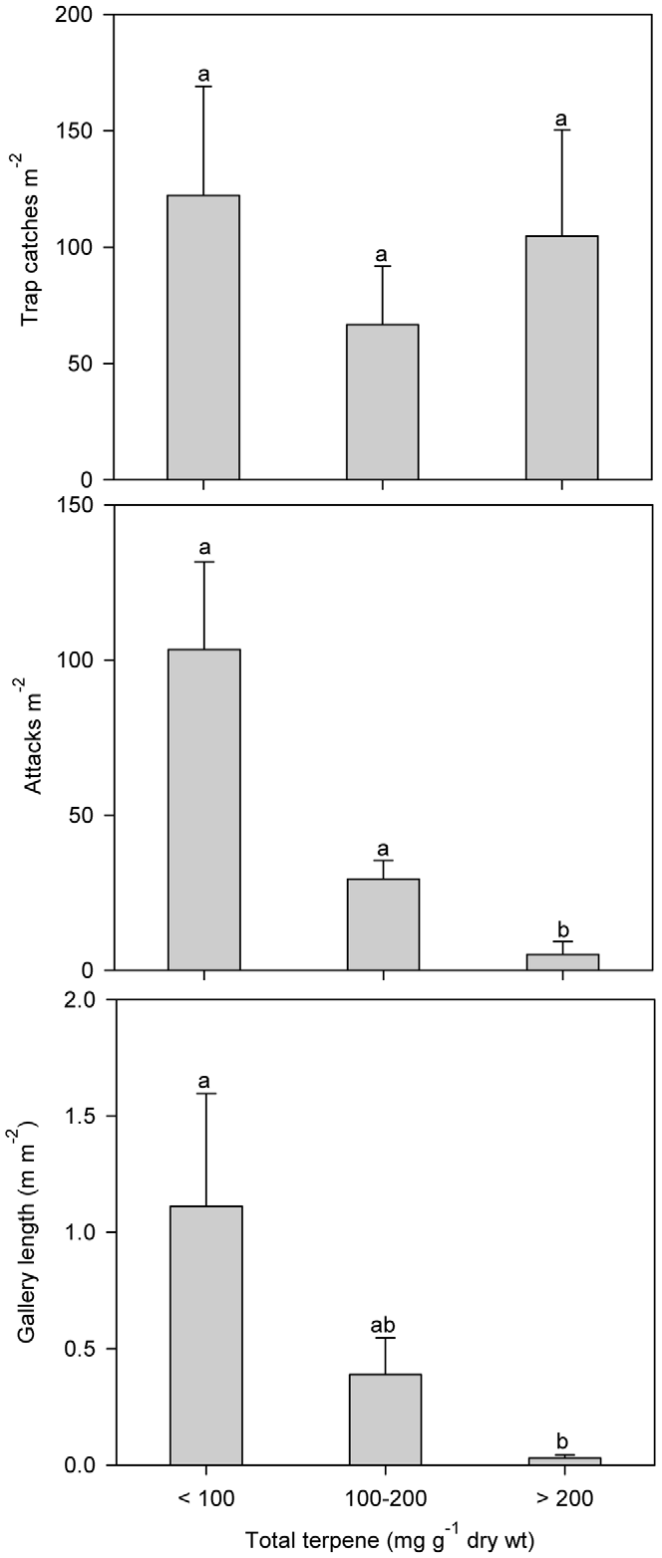
Trap catches, beetle attack density and total gallery length in Norway spruce trees with different induced terpene levels in the bark (<100: n = 6; 100 –**200: n = 7; >200: n = 7).** Chemical samples were taken from the reaction zone 35 days after inoculation with the bluestain fungus *Ceratocystis polonica*. Data are expressed as means + 1 SE. Bars with different letters were significantly different by LSD test at *p* = 0.05 following ANOVA.

To further test the inhibitory effect of terpenes on beetle colonization we compared beetle colonization in trees with different terpene levels in the reaction zone 35 days after inoculation (i.e. low: <100 mg terpene; medium: 100–200 mg terpene, and high: >200 mg terpene g^−1^ dry phloem). Beetle colonization decreased significantly with increasing terpene levels in the bark; trees with high terpene levels had only 28.6% and 34.5% the amount of beetle attacks and gallery length as trees with medium terpene levels, and 4.9% and 2.6% of that in trees with low terpene levels (Fig. 5; F_2,17_ = 0.50, *p* = 0.62 for trap catches, F_2,17_ = 21.53, *p*<0.0001 for attacks, F_2,17_ = 4.56, *p* = 0.026 for galleries).

## Discussion

This study clearly shows that fungal inoculation induces a strong quantitative terpene response in Norway spruce bark and that trees with strong terpene accumulation suffer much less bark beetle colonization than other trees. To the best of our knowledge, this is the first clear demonstration of a dose-dependent inhibitory effect of induced terpene accumulation on tree colonization by bark beetles under field conditions.

Accumulation of terpenes following fungal inoculation is well known in Norway spruce [14] and other conifers [20], [21], [26], [39], [40]. In this study we observed very strong terpene induction in Norway spruce, with total terpene levels increasing 240 fold in some trees 35 days after *C. polonica* inoculation. In Scots pine *Pinus sylvestris* L. monoterpene induction started one day after inoculation and increased up to 800 fold and 30 fold 28 days after *Leptographium wingfieldii* M. Morelet and *Ophiostoma canum* (Münch) H. & P. Sydow inoculation, respectively [26]. This emphasizes that conifer defense reactions are time-dependent dynamic processes, and the speed and intensity of terpene induction depends on the virulence of the challenge, the tree species and/or the physiological status of individual trees.

As is commonly found in conifers we observed large individual variation in induced terpene levels and beetle colonization between experimental trees, probably due to different genetic backgrounds or physiological conditions of the trees [41]. These differences allowed us to correlate induced terpene levels with beetle colonization for individual trees. As expected we found significantly higher terpene levels 35 days after fungal inoculation in resistant trees than in trees that became extensively colonized by bark beetles. This agrees with previous observations from the lodgepole pine *Pinus contorta* Douglas var. *latifolia* Engelmann ­ mountain pine beetle *Dendroctonus ponderosae* Hopkins system [42], [43], where resistant trees on average produced more resin than susceptible trees in response to artificial inoculation with a beetle-vectored fungus. For another bark beetle, the pine engraver *Ips pini* Say, Raffa and Smalley [20] found a positive relationship between terpene concentrations and beetle mortality in lab bioassays using synthetic monoterpene mixtures. We did not find a simple linear correlation between *in vivo* induced terpene levels in Norway spruce and beetle colonization, but there was a highly significant correlation between ranks of terpene levels and beetle colonization. There seemed to be two possible threshold concentrations; one for almost complete exclusion and another for significantly reducing beetle colonization. We thus present solid evidence suggesting that trees with a strong induced terpene response to *C. polonica* inoculation were more resistant to bark beetles.

Terpenes have been demonstrated to be toxic to bark beetles in lab bioassays [36], [44] and to inhibit germination and mycelial growth of the beetle's phytopathogenic fungal associates [37], [45], [46]. In addition, high levels of terpene emission might influence tree resistance indirectly by interacting with the aggregation pheromones that regulate bark beetle host colonization [35], [41], [47]. However, if anything the terpene quantities detected in our trees are most likely to have had positive and not negative effects on *I. typographus* attraction, since monoterpenes are known to synergize the attraction of bark beetles to aggregation pheromones [35]. Thus, the reduced beetle colonization observed in trees with high terpene levels was probably due to physical repellency or chemical toxicity of terpenes and other allelochemicals to the beetles and their associated fungi.

Although terpene induction generally inhibited beetle colonization in a dose-dependent way, a few exceptions were observed. Three trees with low terpene levels (<100 mg terpene g^−1^ dw) had relatively short gallery lengths (Fig. 4). One of these trees had the highest terpene level of all experimental trees 14 days after inoculation, and the other two had high levels of thunbergol at both sampling times, a compound that previously has been shown to correlate with tree resistance against *C. polonica* [30]. Thus, in addition to quantitative effects of terpenes and other allelochemicals on beetle colonization there may also be qualitative effects. Limonene is one compound that has been suggested to be more toxic to bark beetles than other monoterpenes [36], but other studies indicate that beetles are more strongly affected by total monoterpene concentrations than by any particular compound [20]. In our experiment reduced beetle colonization seemed to be due to the collective effects of all terpenes, but some monoterpenes, such as β-phellandrene, myrcene, (-)-limonene, (+)-limonene and camphene were somewhat more closely correlated with beetle colonization than other terpenes (R^2^ = 0.23−0.29 versus R^2^  = 0.0002−0.19).

Terpene levels in the trees 14 days after fungal inoculation showed no correlation to tree resistance to bark beetle colonization, whereas terpene levels at day 35 correlated well with resistance. Since upregulation of terpene biosynthesis in conifers may start within hours or days of an infection [26] it was surprising that terpene induction was most pronounced between 15 and 35 days after inoculation. However, this agrees with previous field experiments demonstrating that Norway spruce requires 2–4 weeks to acquire resistance to bark beetle attack and massive fungal inoculation [27], [28]. The relatively long time span before resistance is achieved may be explained by genetic and/or ecological constraints on these defenses. However, the intense and effective terpene accumulation between 15 and 35 days after fungal infection indicates that up-regulation of terpene-based defense mechanisms is important in our system.

Improved knowledge about tree defenses is needed to understand how ecological disturbances influence interactions between insect and their host trees, and more specifically such knowledge may be useful for developing management strategies that decrease the impact of tree-killing bark beetles. In this study we have demonstrated strong and variable terpene accumulation in Norway spruce trees inoculated with *C. polonica* and shown that *I. typographus* avoids trees with high terpene levels. This is the first demonstration of a dose-dependent effect of terpenes on bark beetle colonization under field conditions, and supports the idea that strong terpene induction may be instrumental in tree resistance against bark beetle attack.

## Materials and Methods

### Field procedures

The experiment was carried out in a pure stand of 48-year-old Norway spruce at Tönnersjöheden Experimental Forest, Halland, Sweden (56° 41′ N, 13°4′ E). Twenty trees (mean diameter at 1.3 m height: 207 mm; range: 161–248 mm) were chosen along a stand edge. On 22 April 2008, the lower part of the stem of each tree (0.8 m–3.8 m above ground) was inoculated with *C. polonica* at a density of 20 m^−2^ bark surface, using a 5 mm cork borer. About ∼30 µl inoculum consisting of mycelium that had been growing on malt agar (2% malt (BD Bacto, BD, USA), 1.5% agar (Apotekproduksjon AS, Oslo, Norway), pH 5.8) for 1 week was used at each inoculation site. The strain used was NFLI 1993 – 208/115, which was isolated from a Norway spruce log inoculated with the bark beetle *Polygraphus poligraphus* L. [48].

Fourteen and 35 days after fungal inoculation, two bark samples for chemical analyses (each consisting of two plugs of outer bark and phloem, one above the other) were taken from each tree using a 5 mm cork borer. One sample was taken immediately above an inoculation hole at ca 1.3 m stem height to quantify induced terpene levels within the reaction zone. Another sample was taken 30 cm below the inoculated stem section (at ca 0.5 m height, where no chemical induction was observed in an earlier similar study [30]) as control.

On 27 May, after the final chemical samples were collected, a 40 cm long pheromone dispenser tape (Hercon® type releasing methylbutenol, *cis*-verbenol and ipsdienol at a ratio of about 160∶7∶1 [50]); was attached at 0.75 m height on a pole standing ∼40 cm from the SW side of each tree to attract bark beetles. At the same time, one sticky trap (Pherobank®, 10×15 cm) was attached to the bark at 1.5 m height on the SW side of each tree. The number of beetles landing on the sticky traps was recorded on 11 and 25 June. According to pheromone trap catches in another stand in the vicinity beetle flight in the area started in late April, peaked around 8 May and had a second smaller peak in early June. On 26 June, the trees were inspected for beetle colonization. Beetle attack density was determined by counting the number of beetle entrance holes on the bark surface within a 0.2 m wide band around the stem (1.7–1.9 m above ground). Beetle tunnelling was determined by carefully exposing three randomly selected attacks within each band with a knife and recording the length of all maternal galleries. Beetle colonization on individual trees was determined as the average of the attack density and total gallery length (both normalized relative to the average for all 20 trees).

### Sample extraction and analyses

The outer cork bark was removed from the bark plugs, and the phloem was submerged in 1.0 ml of hexane containing 0.20 mg pentadecane (Lancaster synthesis, England) as an internal standard and 0.12 mg 3-tert-butyl-4-hydroxyanisole (Fluka, Switzerland) as antioxidant. The samples were extracted in hexane at room temperature for 48 h before the extract was transferred to new vials and kept at –25°C until analyses. The phloem plugs were dried at 80°C for 6 h, and then weighted on a Sartorius electronic balance for absolute amount calculation.

The hexane extracts were separated, identified and quantified using a Varian 3400 gas chromatograph (GC) equipped with a DB-wax capillary column (30 m×0.25 mm×0.25 µm, J&W Scientific, CA, USA) and connected to a Finnigan SSQ 7000 mass spectrometer (MS). A split/splitless injector was used with a 30 s splitless injection at 225°C. The sample (1 µl) was introduced to the injector by a Finnigan A200S autosampler and analyzed by the following temperature program: 40°C for 3 min, increasing to 230°C at a rate of 4°C min^−1^, and remaining constant at 230°C for 19 min. Helium was used as the carrier gas at a flow of 1 ml min^−1^. Electron ionization mass spectra were acquired at 70 eV with the ion source at 150°C and a mass range of 30–400 Da. The enantiomeric composition of α-pinene and limonene was analyzed by a two dimensional-GC system consisting of two Varian 3400 GCs [49]. The first GC was equipped with a DB-wax column (30 m×0.25 mm×0.25 µm, J&W Scientific, CA, USA) for pre-separation and the second GC with a β-cyclodextrin column (J&W Scientific™, 30 m×0.25 mm×0.25 µm) for chiral separation by using a previously described method [30]. The compounds were identified by comparing retention times and mass spectra with available authenticated standards, or by comparing retention indexes and mass spectra with Massfinder 3.0 (Hochmuth Scientific Consulting, Germany) and the reference libraries of NIST (National Institute of Standards and Technology). The amounts of terpenes were calculated relative to the internal standard and expressed as mg g^−1^ dry weight equivalent to the mass of pentadecane.

### Data analysis

One-way ANOVA was used to test for differences in terpene levels between uninoculated stem sections and inoculated stem sections on day 14 and 35. Since there were no significant differences in terpene levels in uninoculated stem sections at day 14 and day 35 (*p* = 0.17−0.99 and F = 0.0002−1.95 for the different terpenes) we used the mean of the two time points as the baseline control level in all analyses. One-way ANOVAs were used to test for differences in beetle attack density or gallery length in trees with different terpene levels, and differences in terpene levels in trees with different levels of bark beetle attack. If treatments were significantly different (*p* <0.05), means were separated using LSD at *p* = 0.05 (Statistica 6.0, Statsoft Inc., USA). Data on terpene quantities were log(y+1) transformed and proportional data were arcsin-transformed before ANOVA to correct for unequal variance and departures from normality.
